# Equivalent Cement Clinker Obtained by Indirect Mechanosynthesis Process

**DOI:** 10.3390/ma13215045

**Published:** 2020-11-09

**Authors:** Rabah Hamzaoui, Othmane Bouchenafa

**Affiliations:** Institut de Recherche en Constructibilité IRC, ESTP, Université Paris-Est, 28 Avenue du Président Wilson, 94234 Cachan, France

**Keywords:** cement clinker, clinkerization, indirect mechanosynthesis, nanostructured materials, crystalline structures

## Abstract

The aim of this work is to study the heat treatment effect, milling time effect and indirect mechanosynthesis effect on the structure of the mixture limestone/clay (kaolinite). Indirect mechanosynthesis is a process that combines between mechanical activation and heat treatment at 900 °C. XRD, TGA, FTIR and particle size distribution analysis and SEM micrograph are used in order to follow thermal properties and structural modification changes that occur. It is shown that the indirect mechanosynthesis process allows the formation of the equivalent clinker in powder with the main constituents of the clinker (Alite C_3_S, belite C_2_S, tricalcium aluminate C_3_A and tetracalcium aluminoferrite C_4_AF) at 900 °C, whereas, these constituents in the conventional clinker are obtained at 1450 °C.

## 1. Introduction

Portland cement is manufactured from raw material obtained by mixing and grinding limestone and minerals rich in silica and alumina (clay or kaolin) and other additives. This mixture is then calcined at 1450 °C to obtain the clinker. The clinker is finely crushed and blended with a source of sulfate (gypsum or anhydrite) and other minerals to form the cement [1].

However, the production of cement is responsible for high energy consumption. For example, in 2014, the cement manufacturer was the third largest industrial energy consumer (7% of the global industrial energy equivalent to 10.7 exajoules) and the second largest quota of total direct industrial CO_2_ emissions (27% equivalent to 2.2 gigatons per year) [2].

To respond to the demand for cement and at the same time avoid damaging more of the planet, it is necessary to find solutions to decrease energy consumption and CO_2_ emission by the clinkerization process. Many solutions were proposed by scientists such as energy efficiency and alternative fuels [2,3,4,5], carbon capture and storage (CCS) [2,3], the substitution of clinker by coproducts [6,7,8,9,10,11], and the substitution of clinker by the activated mineral additions (kaolinite, fly ashes, and slag) carried out by mechanosynthesis [12,13,14].

Mechanosynthesis is described as a high-energy milling process using a ball and/or rod as milling tools [15,16,17,18,19]. In general, mechanosynthesis is defined as a ball milling process, in which the powder mixture is placed with balls in a container, subjecting powders to high-energy collisions provoked by the balls. Mechanosynthesis is characterized by the repetitive welding/fracturing (collage/decollage) movement of the powder mixture. This movement allows one to obtain metastable crystalline or nanocrystalline phases, and to transform crystalline phases into amorphous phases. Nanocrystalline materials obtained by mechanosynthesis have been the subject of much research over the past two decades. As their name suggests, they are single-phase or multiphase polycrystals with a grain (crystallite) size at the nanoscale (1–100 nm). Half of the material by volume consists of grain joints or interphases [20,21,22,23,24].

Mechanosynthesis makes it possible to reduce the size and combine solids from the micrometer scale of a powder particle, while crystallites (the grains forming a particle) are reduced to a nanometric size, resulting in nanostructured materials.

The mechanosynthesis process can be divided into three steps: in the first step, the particles rearrange and stack, the particles slide over each other with a minimum of deformation and fractures, which leads to a particle size reduction and a particle shape change. In the second step, elastic and plastic deformations are applied to the particles. During this phase, the phenomenon of cold welding is most commonly observed, which has the effect of increasing the size of the powder particles due to their agglomeration. In the last step, the particles are fractured, resulting in additional deformation and/or fragmentation of the particles, resulting in a reduction in particle size [20,21,22,23,24].

Mechanosynthesis was firstly used in the 1970s by John Benjamin to produce nickel and iron-based superalloys by oxide dispersion strengthened (ODS) for aerospace applications [25]. Thereafter, the mechanosynthesis process has been developed and enhanced over time.

Three mechanosynthesis processes can be distinguished [26]. The first process is called direct mechanosynthesis, which consists of mechanically activating one or more powders for a fixed period (short or long milling) until the finished product is obtained [12,13,14,15,18,19]. The ODS process is part of the direct mechanosynthesis [25].

The second process is called indirect mechanosynthesis, a technique that combines mechanical activation usually for a short milling time followed by another process. Comparatively to mechanically activated annealing (M2A) [27,28], mechanically activated sintering (MAS) [29,30] and mechanical activation of self-heat-sustaining reaction (MASHS) can be used [31].

The third process is called mechanochemistry, which refers to any process that uses grinding to initiate chemical reactions. The mechanochemistry applications encompass all provoked chemical reactions by a mechanical activation, such as exchange reactions, reduction/oxidation reactions, compounds decomposition, and phase transformations. Mechanochemistry can be considered as a direct mechanosynthesis [13,32] or indirect mechanosynthesis process [33,34].

This work aims to propose an indirect mechanosynthesis process with the purpose of producing an equivalent clinker with a low heat temperature (<1000 °C). In this process, less fossil energy (coke or fuel, etc.) is used in industrial furnaces. However, more electric energy is used due to high energy milling. This energy can be considered to be less polluting, especially if it is from a renewable or nuclear origin. In addition, it should be noted that the study of energy consumption and CO_2_ emission concerning indirect mechanosynthesis is under investigation and will be soon published.

The process of indirect mechanosynthesis begins with the mechanical activation of the limestone and clay mixture and is followed by heating it for 1 h. Firstly, the study of the temperature effect on the structure of the limestone/clay (kaolinite) mixture at different temperature treatments (400, 600, and 900 °C) is carried out. In addition, the milling time effect (5, 10, 15, and 60 min) on the structure of limestone/kaolinite mixture is investigated. Finally, the combination of these two techniques (milling and heating) is effectuated in order to obtain the equivalent clinker.

## 2. Materials and Methods

### 2.1. Raw Materials and Classic Clinker

The limestone used for this research is supplied by OMYA^®^ (Nièvre, France). It is constituted of >97% of calcium carbonate (CaCO_3_) in a calcite structure. The clay used in this work is Proclay^®^ kaolinite produced in the Beaujard site (Poigny, France); it is formed of kaolinite and quartz. Loss on ignition (LOI) of the mixture (LS/C) is 40% and the ratio (CaO/SiO_2_) is 3.15.

For a classic clinker, an industrial clinker from VICAT company (Créchy, France) produced by the current process (thermal clinkerization) is used as a comparison to the equivalent clinker.

The chemical compositions of raw materials and conventional clinkers are investigated by X-ray fluorescence; the results are shown in Table 1.

### 2.2. Methods

#### 2.2.1. Indirect Mechanosynthesis for the Equivalent Clinker Production

Two processes are used to produce clinker: a high energy ball milling (mechanical activation) and heat treatment.

**Figure 1:**
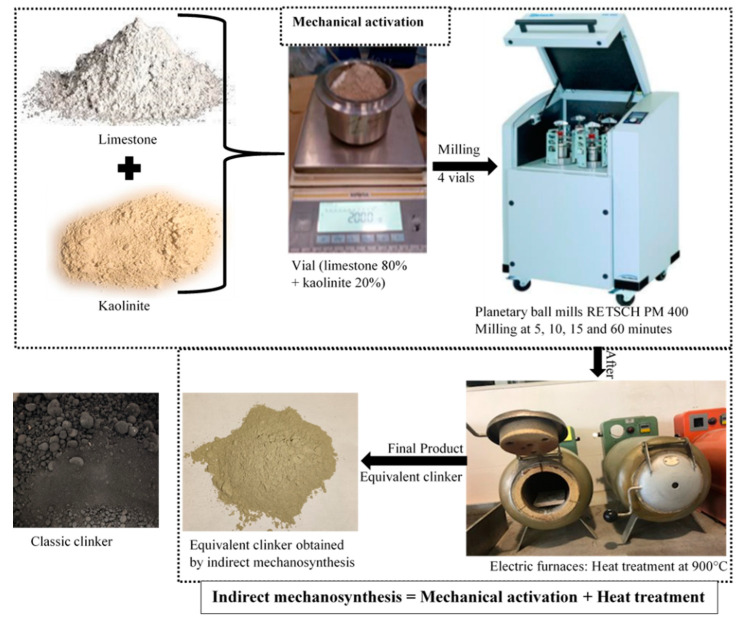
Representative schematic of indirect mechanosynthesis.

Mechanical activation is achieved by using a planetary ball mill (PM 400, RETSCH, Haan, Germany) for the high energy ball milling (see Figure 1). The device consists of four vials mounted on a planar disc. When the disc rotates, the vials move in a circle and in the reverse direction to the disc. The ball/powder weight ratio applied is 4.

To prepare the equivalent clinker, 80% of limestone (LS) (160 g) and 20% clay (C) (40 g) are put in the vial. In this process, steel balls with a diameter of 30 mm and a steel bottle with a capacity of 500 mL are used. To avoid contamination, the vials are well closed. The rotational speed of the disc and the vials are Ω = 400 rpm and ω = 800 rpm, respectively. Different milling times are achieved, between 5 and 60 min. For the thermic process, several temperatures ranging from 400 °C to 900 °C are applied for 60 min using an industrial electric furnace (Solo Swiss SA, Porrentruy, Switzerland) (see Figure 1).

#### 2.2.2. Characterization of Powders

The chemical composition of all materials is determined by an elemental analysis performed by a Bruker S2 RANGER (XRF, BRUKER, Karlsruhe, Germany). For the structural investigation, Bruker D2 PHASER diffractometer (BRUKER, Karlsruhe, Germany) with a continuous scanning mode and Cu Kα radiation (λ = 0.1541 nm) is used. The parameters of the XRD analysis were: 2θ range of 5–70°, an increment of 0.02° for 0.15 s. For the evaluation of the X-ray diffraction pattern, the software DIFFRAC.EVA (BRUKER, Karlsruhe, Germany) with ICDD PDF2 (Newtown Square, PA, USA) is used. Winnel software is used for X-ray patterns fitting to calculate the lattice parameter changes. This fitting takes into account the shift of the high-angle diffraction line for all X-ray patterns using Bragg’s law, Equation (1):

(1) 2dSin(θ)=nλ

where (d): the distance between the planes in the atomic lattice; (θ): the angle between the incident ray and the scattering planes; (n): an integer determined by the order given (n = 1); (λ): wavelength of the incident wave. The average lattice parameter (a) is determined for all X-ray patterns from different angles at lines (200), (220), and (201) of the calcium oxide structure by using Equation (2):

(2) $$d=\sqrt{\frac{a^{2}}{h^{2}+k^{2}+l^{2}}}$$

where (hkl) are Miller indices associated to different angles; (a) is lattice parameters. The accuracy of the above expressions is evaluated by applying the standard deviation to all lattice parameters. The analysis of all X-ray patterns indicates that the precision of the lattice parameter evaluation is less than 1 × 10^−4^ nm.

Using the XRD profile analysis, we calculated the crystallite size (D) and the lattice strain by the Williamson–Hall method [35], as shown in Equation (3):

(3) $$\beta =2\varepsilon \tan\left(\theta \right)+\frac{0.9\lambda }{D\;\cos\left(\theta \right)}$$

where (β) represents the full width at half maximum (FWHM) obtained with Winnel software; (θ) is the Bragg’s angle; (λ) is the X-ray radiation wavelength; (ε) is the effective lattice strain; (D) is the average crystallite size. Equation (4) can be rearranged as:

(4) $$\beta \cos\left(\theta \right)=2\varepsilon \sin\left(\theta \right)+\frac{0.9\lambda }{D}$$

For all peaks of the same structure, the plot of βcos(θ) of each peak versus sin(θ) of each peak is achieved and a straight line is obtained. The straight-line intercept represents (0.9 λ)/D and the straight-line slope represents 2ε. From these, the crystallite size D and lattice strain ε are evaluated.

To determine thermal behavior, TG analysis by a TGA/DSC 2 instrument (Mettler–Toledo, Viroflay, France) with a heating speed of 20 °C/min beginning from 25 °C to 1000 °C is accomplished. To prevent contaminating the sample with the environment, nitrogen is used as purge gas (40 mL/min).

FTIR spectroscopy is performed by a Nicolet IS10 instrument (Thermo Scientific, Waltham, MA, USA), with a Smart iTR accessory (with Diamond Plate, Thermo Scientific, Waltham, MA, USA). With an ATR (attenuated total reflectance) accessory, FTIR spectroscopy can examine solids, liquids, pastes, and gels. The results are evaluated using Omnic software (v2.0, Thermo Scientific, Waltham, MA, USA).

The particle size distributions are measured with a granulometer laser LS 13 320 XR from BECKMAN COULTER (Villepinte, France). Liquid sample dispersion method with a size distribution between 0.01 and 2000 μm is used.

In this study, the liquid used was ethanol to prevent the hydration of the samples studied. The liquid sample dispersion method is coupled with its enhanced PIDS (polarization intensity differential scattering) technology and a wide measuring range offering higher resolution, greater accuracy and reproducible results. For the dispersion of the particles, soundwaves to break up aggregates are used and a few milligrams of product in a volume of ethanol are put and agitated for at least 2 min with a magnetic agitator.

The morphology of unground powders and clinker powders A and B is analyzed by a scanning electron microscopy (SEM) S-3400 (HITACHI, Ibaraki, Japan) with an energy-dispersive spectrometer (EDS) (HITACHI, Ibaraki, Japan).

## 3. Results and Discussions

The results of this work are presented in three parts, the first of which concerns the heat treatment effect on the limestone/kaolinite mixture. The second part shows the milling time effect of direct mechanosynthesis on the limestone/kaolinite mixture. The third part discusses the indirect mechanosynthesis effect on this mixture. In this part, the formation of the equivalent clinker is obtained.

### 3.1. Heat Treatment Effect on LS/C Mixture

**Figure 2:**
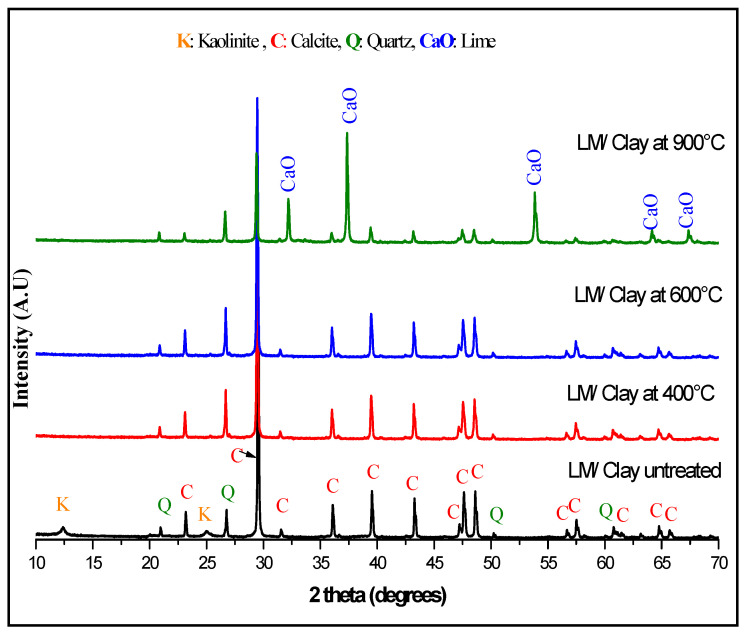
X-ray diffraction patterns of untreated limestone/clay mixture and heat-treated mixture at different temperature.

Figure 2 presents the XRD patterns of the untreated limestone/clay mixture (LS/C) and (LS/C) treated at 400, 600 and 900 °C. For the untreated (LS/C) mixture, the presence of crystalized rhomboidric calcite (PDF#01-083-1762) is observed. In addition, triclinic kaolinite (PDF#00-003-0058) and hexagonal quartz (PDF#00-046-1045) are found.

Concerning the patterns of the heat-treated (LS/C) mixture, it is remarked the disappearance of all kaolinite peaks up to a temperature of 400 °C. This structure modification can be due to the dehydroxylation of kaolinite according to the following formula (5) and the formation of the amorphous metakaolinite structure Al_2_O_3_·2SiO_2_ [15,35,36,37]:

Al_2_Si_2_O_5_(OH)_4_ → Al_2_O_3_·2SiO_2_ + 2H_2_O (5)

During heating, kaolinite starts to lose water at about 400°C, and dehydration is almost complete at about 525 °C [35]. Kaolinite dehydration depends on particle size, crystallinity, and purity [13,35,36].

Concerning limestone, it has been found that heat treatment has provoked partial decomposition resulting in the decaying of calcite peaks as function of a heat treatment of 400, 600 and 900 °C. These decaying peaks appeared to decrease in intensity and their tight peaks. In addition, the disappearance of some of the calcite peaks in the positions 2θ: 31.46 °C (0 0 6), 64.71 °C (3 0 0), and 65.67 °C (0 0 12) at 900 °C has been remarked. The disappearance of these calcite peaks can be due to the decarbonation reaction (6) as reported by several researchers [37,38]:

CaCO_3_ → CaO + CO_2_ (6)

**Figure 3:**
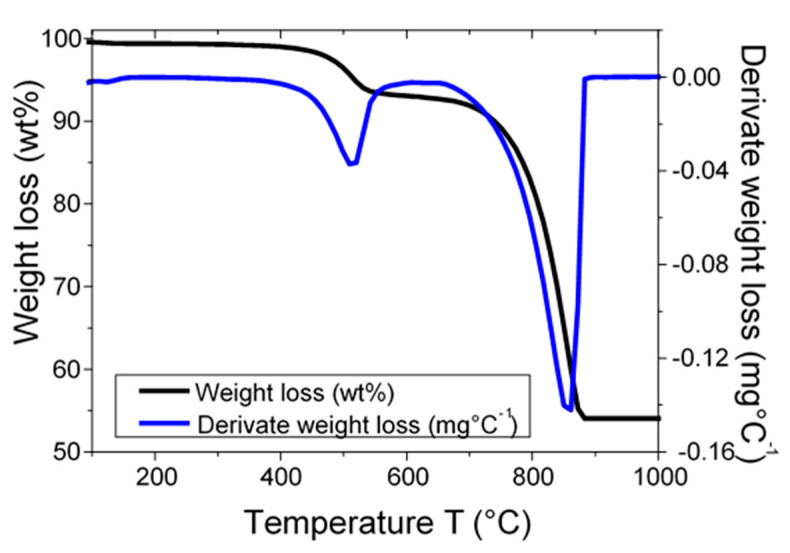
TG (thermogravimetry) and DTG (derivative thermogravimetry) curves of limestone/clay (LS/C) mixtures.

This reaction produces a cubic lime (CaO) (PDF#00-002-1088) which has been observed after a heat treatment of 900°C. Generally, the production of lime is obtained up to 800 °C [37,38,39]. From these observations of CaO formation, a temperature of 900 °C for the thermal treatments for the clinkerization process by indirect mechanosynthesis is chosen. To confirm different decompositions obtained by heat treatment, TG (thermogravimetry) and DTG (derivative thermogravimetry) analyses are performed in the temperature range 25–1000 °C for the thermal behavior of the mixture (LS/C) and shown in Figure 3.

**Figure 4:**
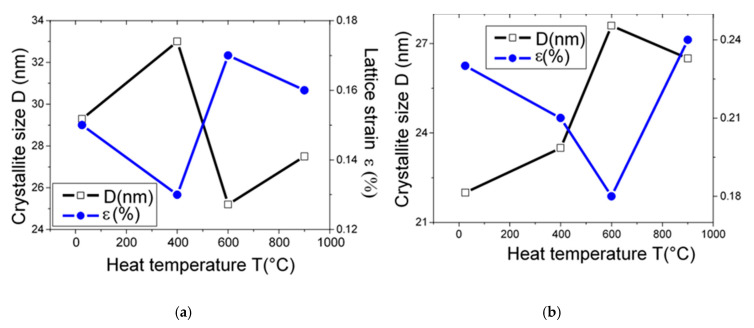
Crystallite size and lattice strain evolution as function of heat temperature: (**a**) quartz, (**b**) calcite.

TG analysis suggests two weight loss regions where the first region of decomposition is bounded between 395 °C and 580 °C, attributed to the dehydroxylation of kaolinite. The second decomposition stage is between 680 °C and 895 °C and is attributed to the decomposition of calcite. The DTG analysis shows the presence of two peaks at 520 °C and 861 °C. To analyze the structural changes induced by heat treatment, the evolution of the crystallite size (D) and the lattice strain (ε) as a function of the heat treatment temperature for both quartz and calcite are shown in Figure 4a,b. Both quantities are given by the instrumental width of the X-ray diffraction peaks.

Concerning the evolution of the quartz structure (Figure 4a), it is observed that the increase in the crystallite sizes is accompanied by the reduction in the lattice strain level when the temperature of heat treatment increases to 400 °C. The value of crystallite size (D) increases from D = 29.3 ± 1.1 nm to 33 ± 1.1 nm for 400 °C, decreases to 25.2 ± 1.1 nm at 600 °C, and has a slight increase to 27.5 ± 1.1 nm for 900 °C.

However, the internal strain value decreases from 0.15 ± 0.01% to 0.13 ± 0.01% for 400 °C and increases to 0.17 ± 0.01% at 600 °C with a slight decrease to 0.16 ± 0.01% when the heat temperature is up to 900 °C. The increase in crystallite size with the decrease in the lattice strain at 400 °C may be due to grain expansion and relaxation of residual stress.

The variation in crystallite size and lattice deformation of the calcite structure as a function of heat temperature is presented in Figure 4b. It is observed that the crystallite size (D) increases from 22 ± 1.1 nm at room temperature to 27.6 ± 1.1 nm at 600 °C with a slight decrease to 26.5 ± 1.1 nm at 900 °C. Concerning the lattice strain evolution, it has been found that the lattice strain value decreases from 0.23 ± 0.01% at room temperature to 0.18 ± 0.01% at 600 °C and increases to 0.24 ± 0.01% at 900 °C. This increase in lattice strain at 900 °C may be attributed to the decomposition of calcite peaks in order to form CaO peaks by decarbonation phenomenon.

Kohobhange et al. [40] have investigated the heat treatment effect on calcium carbonate decomposition by using high-temperature X-ray powder diffraction (HT-XRD). The authors [40] have found that the low lattice strain value for calcite is recorded before thermal decomposition at 675 °C, whereas with the progressive heating at 775 °C, maximum lattice strain value is observed. The authors have attributed this increase to the strong lattice vibrations and atomic displacements over the transformation phase and decomposition from calcite structure trigonal to calcium oxide cubic structure formation. In addition, the authors have remarked that the maximum calcite crystallite size is observed at 775 °C [40].

### 3.2. Milling Time Effect on Mixture LS/C

**Figure 5:**
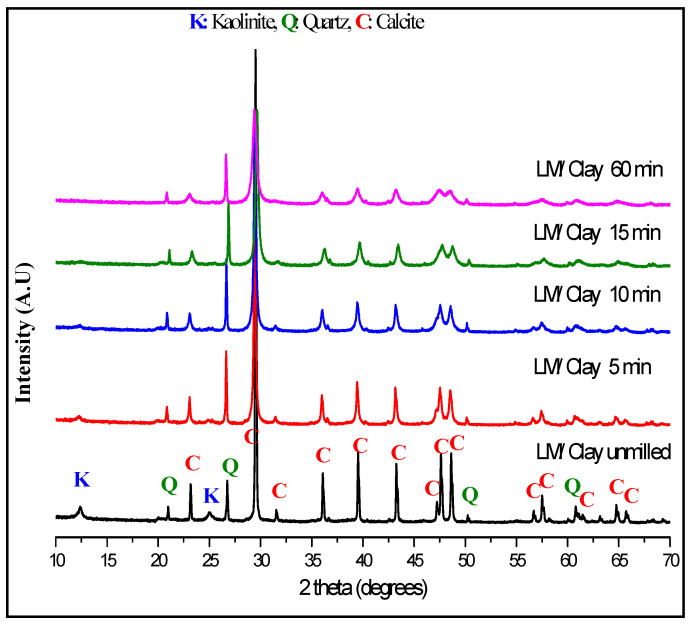
Diffraction patterns of a raw and milled mixture of limestone/clay for different duration.

Figure 5 presents X-ray diffraction patterns of the milling time effect on the structure of (LS/C) mixture.

Concerning the kaolinite structure, we found the decaying of peaks at 2θ = 12.53° K (001), 2θ = 25.17° K (002) for 5 min and 10 min of milling. The total disappearance of theses peaks is obtained after 15 min of milling which reaches complete amorphization. For quartz structure, we observed the peaks enlargement as function of milling time which may be due to the crystallite size reduction and formation of nanostructured quartz [18,21,24,25].

Hamzaoui et al. [13] have studied the milling time effect on the structure and thermal behavior of proclay kaolinite by using a planetary ball mill. The authors have obtained an amorphous structure of kaolinite followed by nanocrystalline quartz formation after one hour of milling. The transition from the crystalline state into an amorphous state can be produced by internal strain accumulation as defects (point defects, dislocations, etc.) in the crystalline material after deformations provoked by ball milling. These defects increase the Gibbs free energy of the crystalline phase. When the high defect density in the crystalline material increases and Gibbs free energy reaches the amorphous phase level, the transition from the crystalline phase to the amorphous phase can be carried out [16,20,24].

Concerning calcite peaks, it is remarked that the peak at 2θ = 31.46 °C (006) disappears completely after 15 min of milling. For the other calcite peaks, the reduction in their intensities, even for the most intense peak at 2θ = 29.42 °C (104), can be observed, as well as peak enlargement as a function of the milling time increase, which suggests nanostructured calcite formation.

**Figure 6:**
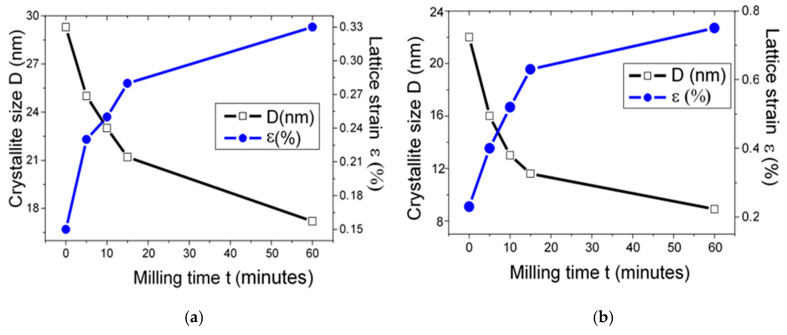
Crystallite size and lattice strain evolutions as function of the milling time of: (**a**) quartz, (**b**) calcite.

Figure 6a,b shows the changes of the crystallite size (D) and the lattice strain (ε) versus the milling time for both quartz and calcite.

It is observed that the crystallite size decreases, and lattice strain increases versus milling time for both quartz and calcite structure. Concerning quartz structure (Figure 6a), it has been found that the crystallite size decreases from 29.2 ± 1.1 (nm) to 17.2 ± 1.1 (nm) after 60 min of milling. The lattice strain of quartz increases from 0.15 ± 0.1 (%) to 0.33 ± 0.1 (%) after 60 milling time. The decrease in crystallite size and the increase in lattice strain can be attributed to a high-energy milling process that uses balls in which powder particles are subjected to the transfer of mechanical energy to the powder particles by plastic deformation.

Kohobhange et al. [41] have studied the effect of prolonged milling until 360 h of conventional ball milling on modification quartz structure. The authors have remarked that, versus milling time, an increase in lattice strain and dislocation density followed by a fast decrease in crystallite size can be observed.

Figure 6b presents the changes of the crystallite size and lattice strain of the calcite structure versus milling time. It is observed that crystallite size decreases from 22 ± 1.1 nm to 8.9 ± 1.1 nm and the lattice strain increases from 0.23 ± 0.01 (%) to 0.75 ± 0.01 (%) when the milling time reaches 60 min. In comparison to heat treatment, it is observed that the lattice strain of both (quartz and calcite) is more important in the milling process. This mechanical transfer introduces strains into the powder through the dislocation generation and other defects. These defects provoke a reduction in grain sizes, generally produced as a consequence of the reduction in the diffusion distances. All these effects lead to distinct structural features that affect the final product and the blended elemental powders during the milling process. Among these properties is the formation of the nanostructured material with the stable state at room temperature.

### 3.3. Indirect Mechanosynthesis and Equivalent Clinker Formation

#### 3.3.1. XRD

**Figure 7:**
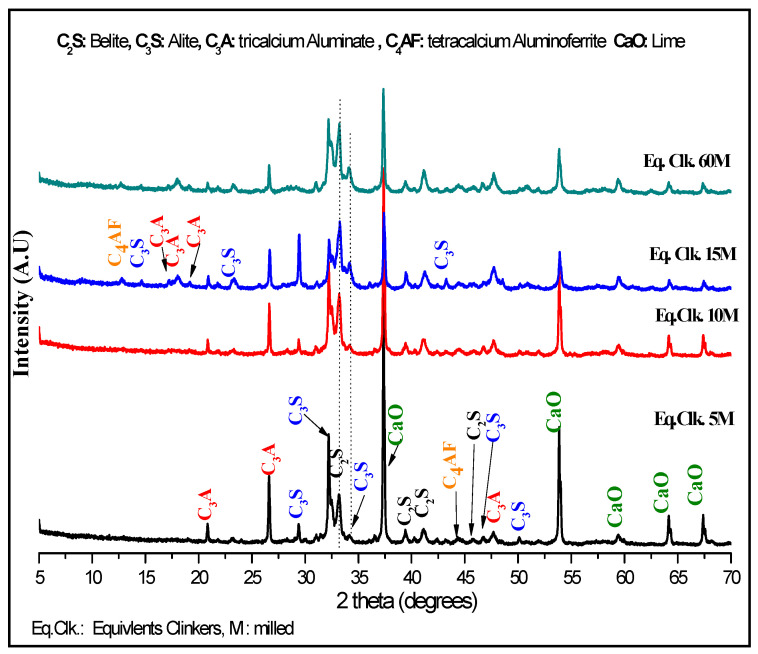
X-ray diffraction patterns of the different clinkers produced by mechanosynthesis.

X-ray patterns of the (LS/C) mixture milled at a different time (5, 10, 15 and 60 min) followed by a heat treatment at 900 °C for 1 h is presented in Figure 7. It is observed that the indirect mechanosynthesis process allows one to obtain the main constituents of the clinker (C_3_A, C_2_S, C_3_S, C_4_AF).

Peaks of a C_3_A (tricalcium aluminate Ca_3_Al_2_O_6_) structure have been found in the positions 2θ: 20.97° (2 3 0), 26.76° (4 2 1), 47.66° (8 0 0) in all X-ray diffraction patterns of the equivalent clinkers (eq. clk.). In addition, the additional peaks are identified in the 2θ positions 14.20° (2 1 1), 17.42° (2 2 1), 19.29° (3 1 1) for the mechanically activated eq. clk. of 15 and 60 min and 1 h of heating at 900 °C.

Concerning peaks of an Alite C_3_S (tricalcium silicate Ca_3_SiO_5_) structure, they have been found for all X-ray diffraction patterns in different 2θ positions: 29.42° (3 2 $\bar{2}$) (not found in eq. clk. 60 min), 30.07° (6 0 2), 43.07° (9 2 $\bar{4}$), 46.82° (4 2 $\bar{2}$), 50.01° (3 2 2). For the eq. clk. 15 M and 60 M, some additional peaks have been identified in the 2θ positions: 14.90° (0 1 1) and 22.95° (6 1 $\bar{1}$). However, it is remarked that the intensity of the peak at 2θ 29.42° is higher with a longer milling time except for a milling duration of 60 min.

The 2θ positions: 32.83° (3 0 0), 39.58° (3 2 1), 41.24° (0 4 2), and 45.82° (2 1 4) reveal the existence of peaks of Belite C_2_S (dicalcium silicate Ca_2_SiO_4_) with the higher intensities for longer milling time.

It is noticed that the presence of calcium oxide (CaO) with a cubic structure in all equivalent clinkers produced by indirect mechanosynthesis is observed. Several peaks of CaO cubic structure are identified in the 2θ positions: 37.38° (2 0 0), 53.90° (2 2 0), 64.21° (3 1 1), 67.43° (2 2 2). Additionally, it is remarked that the lower the intensity peaks, the higher the milling time. Using TOPAS software in order to quantify the amount of CaO, we found 15%, 13%, 11% and 9% for 5, 10, 15 and 60 min of milling, respectively. It is observed that the lower the CaO structure, the higher the milling time. It is noted that the main objective of this study is to show the feasibility of indirect mechanosynthesis for obtaining the different constituents of clinker (C_2_S, C_3_S, C_3_A, and C_4_AF) with the (LS/C) mixture.

To understand the evolution of calcium oxide versus milling time, the evolutions of the lattice parameter, crystallite size, and lattice strain by using instrumental position 2θ and width of the X-ray diffraction peaks are followed.

**Figure 8:**
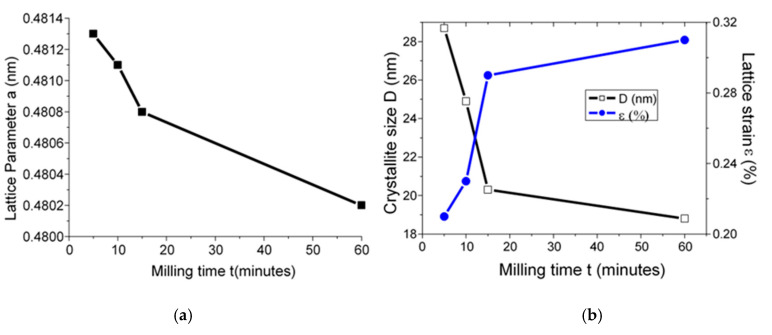
Evolution at 900 °C of CaO versus milling time t (minutes) of: (**a**) lattice parameter (nm), (**b**) crystallite size and lattice strain.

Figure 8a presents the change of lattice parameter versus milling time for calcium oxide formation.

It is observed that the lower the lattice parameter (a) (nm), the higher the milling time from 0.4813 ± 0.0001 nm at 5 min of milling to 0.4802 ± 0.0001 nm. The evolution of crystallite size and the lattice strain of calcium oxide formation versus the milling time are shown in Figure 8b. It has been found that the lower crystallite, the higher the lattice strain with the higher milling time. The decrease in crystallite size value is from 28.7 ± 1.1 nm at 5 min of milling to 18.8 ± 1.1 nm at 60 min of milling. The increase in lattice strain value is from 0.21 ± 0.01% at 5 min of milling to 0.31 ± 0.01% at 60 min of milling.

**Figure 9:**
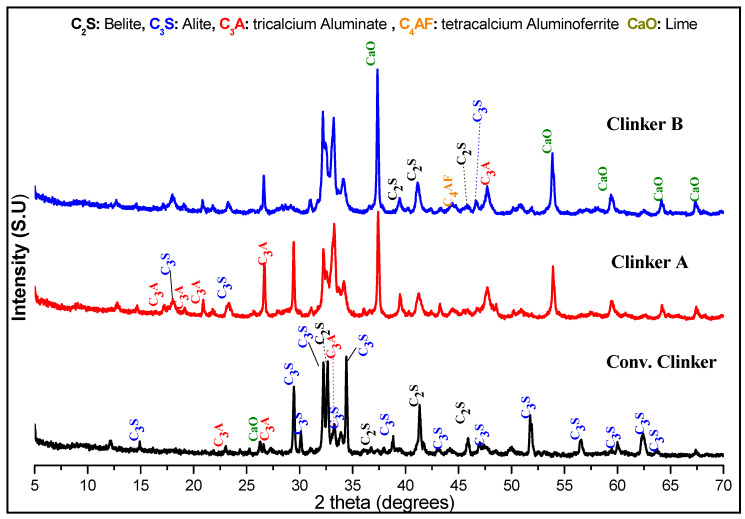
X-ray diffraction patterns of clinker A and B produced by mechanosynthesis versus conventional clinker.

XRD pattern comparison between the equivalent clinker and the conventional clinker obtained by calcination or clinkerization from VICAT cited in our reference [42] is shown in Figure 9.

The X-ray diffraction patterns of conventional clinker from VICAT (Conv clinker), equivalent clinker 15 min of milling + 1 h at 900 °C of heating (clinker A) and equivalent clinker 60 min of milling + 1 h at 900 °C of heating (clinker B) are presented. It is observed that with indirect mechanosynthesis, different crystalized phases, such as alite (C_3_S), belite (C_2_S), aluminate tricalcium (C_3_A), and tetracalcium aluminoferrite (C_4_AF) at heat treatment of 900 °C, are produced.

Generally, clinkers are manufactured by heating a mixture of limestone and clay, or similar bulk materials of similar composition and adequate reactivity, to a final temperature of about 1450 °C. They contain 55–65% of the Alite. It is the most important constituent of all clinkers, the Alite; C_3_S is tricalcium silicate (Ca_3_SiO_5_) and formed between 1300–1450 °C. Belite C_2_S constitutes 15–30% of normal clinkers. It is obtained between 1000 and 1300 °C for normal clinkers. For tricalcium aluminate, C_3_A (Ca_3_Al_2_O_6_) constitutes 8–12% of most normal clinkers. The tricalcium aluminate C_3_A is obtained after the formation of the liquid phase for temperatures between 1200 and 1400 °C and crystallizing from the melt by the fast air cooling. Tetracalcium aluminoferrite C_4_AF(Ca_2_AlFeO_5_) makes up 5–15% of normal clinkers. It is formed at 1200–1400 °C and after an air cooling [1,5,43].

#### 3.3.2. FTIR

**Figure 10:**
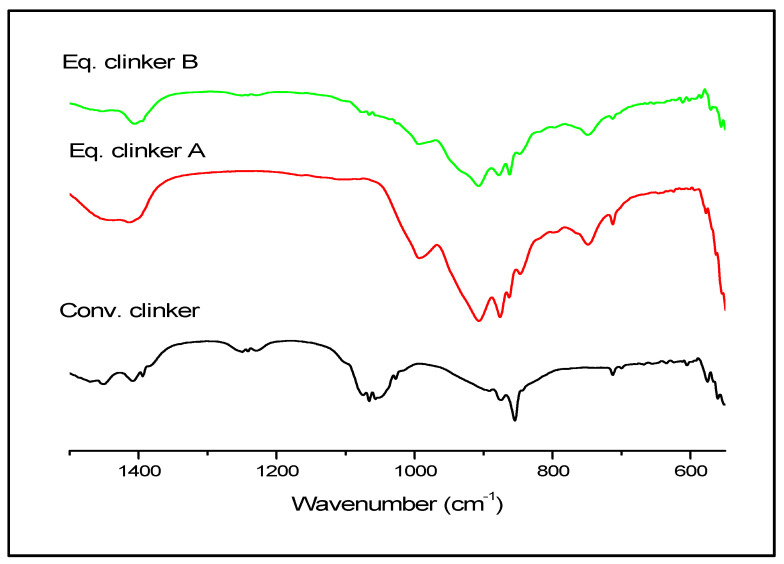
FTIR spectra of the conventional clinker and the clinkers A and B produced by mechanosynthesis.

For the purpose of verifying and confirming the presence of C_2_S, C_3_S, C_3_A, and C_4_AF, FTIR spectroscopy analysis data are given in Figure 10 and Table 2.

The presence of some compounds found in the X-ray diffraction, such as the ß-C_2_S or C_3_S by the absorption band Si-O at 992, 912, and 846 cm^−1^ for the two equivalent clinkers, A and B, is confirmed. Besides, the absorption band Si-O for the conventional clinker is found at 1025 cm^−1^ [44,45]. The width band between 1360 and 1568 cm^−1^ can be related to calcium carbonate (C-O) and the absorption band at 876 cm^−1^ is observed only in two patterns of the two equivalent clinkers, A and B, whereas the band at 712 cm^−1^ is observed in three patterns. Concerning C_3_A identification, we observed an adsorption band (Al-O) at 861 cm^−1^ in two patterns of the equivalent clinkers, A and B. Additionally, for the C_4_AF, a band (Fe-O) at 700 cm^−1^ is found and only observed in the conventional clinker [44,45]. It is noticed that more bands are cited in our reference [42].

#### 3.3.3. Granulometry

**Figure 11:**
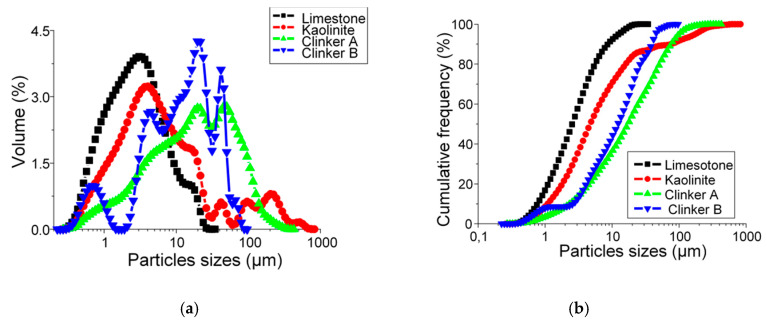
Particle size distribution analysis of raw limestone, raw kaolinite, clinker A and clinker B powders: (**a**) volume (%), (**b**) cumulative volume (%).

To study the granularity and morphology of different powders, laser granular and SEM are used. For raw limestone, raw clay, clinker A and clinker B powders, the particle size distribution (PSD) is shown in Figure 11a,b.

In Figure 11a, it is observed that the raw limestone has modal distribution with maximum peak found at 3.4 μm. Raw kaolinite has a multimodal distribution with four particle size ranges. The first particle size range is meaningfully distinct with a large volume corresponding to the fine particle population with typical sizes between 0.3 μm and 28.7 μm and a maximum peak found at 4 μm. The second range is between 31.5 μm and 60.5 μm with a maximum peak at 41.5 μm. The third range refers to particle sizes from 66.5 μm to 140.1 μm and with a maximum peak at 96.5 μm. The last particle size range is recorded between 140.3 μm and 460 μm with a maximum peak at 223.4 μm. For both raw materials, limestone and kaolinite, the fine particles are predominating. In the literature, the particle size distribution of raw limestone is reported to have unimodal distribution [46] and raw kaolinite (kaolin) is reported to have unimodal, bimodal, and multimodal distribution [14].

Concerning clinker A and clinker B particle size distributions, it has been found that clinker A has a bimodal distribution with the first maximum peak at 19.8 μm and the second maximum peak at 50.3 μm, whereas it is has been remarked that clinker B has multimodal distribution with four particle size ranges. The first particle size range corresponds to the fine particle population with typical sizes between 0.3 μm and 1.5 μm and a maximum peak found at 0.7 μm. The second range is between 1.9 μm and 6.5 μm with a maximum peak at 4.5 μm. The third range is bounded from 6.5 μm to 31.5 μm and with a maximum peak at 21.7 μm. The fourth particle size range is recorded between 31.5 μm and 88 μm with a maximum peak at 41.7 μm. The presence of a multimodal distribution of clinker B can be attributed to the long milling time (60 min) in comparison to clinker A. According to Figure 11b, concerning the particle size distribution cumulative volume, D10, D50, and D90 values of limestone, kaolinite, clinker A and clinker B are summarized in Table 3.

#### 3.3.4. SEM

**Figure 12:**
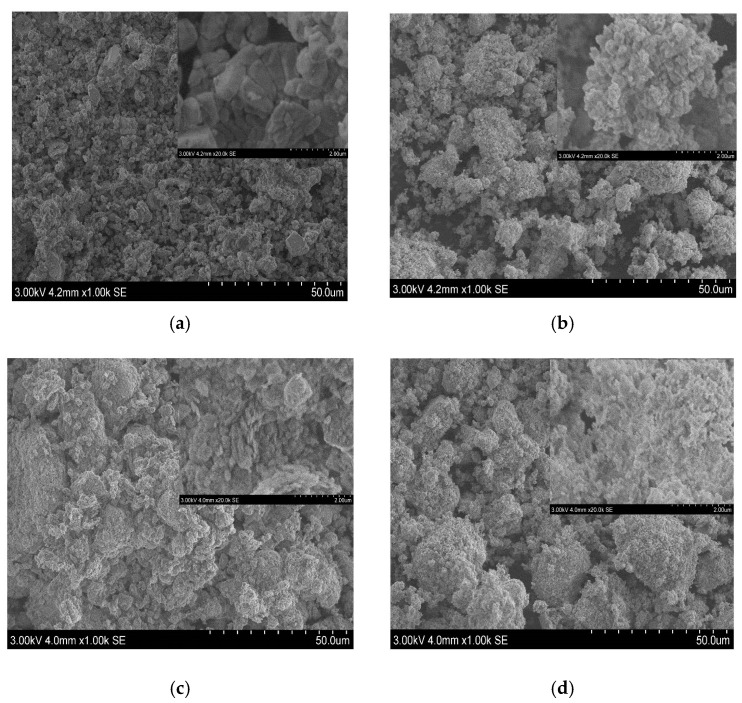
SEM images of: (**a**) raw limestone, (**b**) raw kaolinite, (**c**) clinker A, (**d**) clinker B.

Figure 12a–d present SEM micrographs of raw limestone, kaolinite, clinker A and clinker B powders.

The morphology of raw limestone powder (Figure 12a) is irregular with different small particle magnifications. In addition, it can be seen through particle magnification, stacks of rhombohedral crystals, which are found to be equally distant from one another, forming these small aggregates with the average particle size estimated from SEM pictures to be 3 µm. This result is in concordance with the particle size distribution of limestone.

The morphology of the raw kaolinite is shown in Figure 12b. The particles mostly form stacks and flat particles with irregular shapes. Additionally, different particle sizes can be distinguished, which explains the presence of a multimodal particle distribution with four typical populations.

The morphology of clinker A and clinker B powders (Figure 12c,d) is very similar where the agglomeration of the particles with irregular shapes are observed. Notably, most of the particles have roughhouse surfaces. The average particle size found from different SEM pictures is 20 µm and 17 µm for clinker A and clinker B, respectively.

## 4. Conclusions

In this work, the effect on the structure of the limestone/clay (kaolinite) mixture versus different temperature treatments (400, 600, and 900 °C) for 1 h, milling time (5, 10, 15 and 60 min) and indirect mechanosynthesis process is investigated. Concerning heat treatment, the structural study shows the disappearance of all kaolinite peaks from 400 °C with the formation of metakaolinite structure. In addition, there is the disappearance of some calcite peaks and the apparition of calcium oxide peaks at 900 °C.

Concerning milling time effect on structure, we observed peak enlargement of quartz and calcite as function of milling time.

For the indirect mechanosynthesis process, the formation of the main constituents of the clinkers (C_3_A, C_2_S, C_3_S, C_4_AF) at 900 °C is shown.

Indirect mechanosynthesis is a technique that can achieve the production of clinkers at 900 °C and less, whereas the classical technique for normal clinker is obtained at 1450 °C of calcination.

## Figures and Tables

**Table 1:** Chemical composition of the raw materials and classic clinker.

Oxide	Limestone	Kaolinite	Clinker
CaO	96.79	0.50	71.85
SiO_2_	0.97	69.37	14.66
Al_2_O_3_	0.63	24.08	3.02
FeO_3_	0.22	1.54	3.97
Na_2_O	-	-	-
SO_3_	0.14	0.30	2.26
MnO	0.08	-	-
SrO	0.21	-	0.28
TiO_2_	-	3.52	0.27
P_2_O5	-	0.72	0.24
MgO	0.50	0.60	1.80
K_2_O	0.16	-	1.3
Others	0.42	1.09	0.35

**Table 2:** Assignment of FTIR peaks of clinkers.

Crystalline Phases	Wavenumber (cm^−1^)
ß-C_2_S ou C_3_S (Si-O)	846, 912, 992, 1025, 1025, 1250
CaCO_3_ (C-O)	712, 880, 1400, 1450
C_3_A (Al-O)	861
C_4_AF (Fe-O)	700

**Table 3:** Median particle sizes: D10%, D50% and D90% of raw limestone, raw kaolinite, clinker A and clinker B powders.

Samples	D10 (µm)	D50 (µm)	D90 (µm)
Limestone	0.8	2.6	9.1
Kaolinite	1.0	4.6	78.2
Clinker A	2.4	17.5	79.7
Clinker B	2.6	11.9	40.3

## References

[1] Kurdowski W. (2013). Cement and Concrete Chemistry.

[2] IEA (2018). Technology Roadmap—Low-Carbon Transition in the Cement Industry. www.wbcsdcement.org.

[3] Schneider M., Romer M., Tschudin M., Bolio H. (2011). Sustainable cement production-present and future. Cem. Concr. Res..

[4] Scrivener K.L. (2014). Special Issue—Future Cements Options for the Future of Cement. http://www.lc3.ch/wp-content/uploads/2014/09/0851_ICJ_Article.pdf.

[5] Gartner E. (2004). Industrially interesting approaches to “low-CO_2_” cements. Cem. Concr. Res..

[6] Siddique R., Khan M.I. (2011). Supplementary Cementing Materials.

[7] Payá J., Monzó J., Borrachero M.V., Peris E., González-López E. (1997). Mechanical treatments of fly ashes. Part III: Studies on strength development of ground fly ashes (GFA)—Cement mortars. Cem. Concr. Res..

[8] Payá J., Monzó J., Borrachero M.V., Peris-Mora E., Amahjour F. (2000). Mechanical treatment of fly ashes—Part IV. Strength development of ground fly ash-cement mortars cured at different temperatures. Cem. Concr. Res..

[9] Behim M., Cyr M., Clastres P. (2011). Physical and chemical effects of El Hadjar slag used as an additive in cement-based materials. Eur. J. Environ. Civ. Eng..

[10] Kourounis S., Tsivilis S., Tsakiridis P.E., Papadimitriou G.D., Tsibouki Z. (2007). Properties and hydration of blended cements with steelmaking slag. Cem. Concr. Res..

[11] Scrivener K., Martirena F., Bishnoi S., Maity S. (2018). Calcined clay limestone cements (LC3). Cem. Concr. Res..

[12] Bouchenafa O., Hamzaoui R., Bennabi A., Colin J. (2019). PCA effect on structure of fly ashes and slag obtained by mechanosynthesis. Applications: Mechanical performance of substituted paste CEMI + 50% slag/or fly ashes. Constr. Build. Mater..

[13] Hamzaoui R., Muslim F., Guessasma S., Bennabi A., Guillin J. (2015). Structural and thermal behavior of proclay kaolinite using high energy ball milling process. Powder Technol..

[14] Hamzaoui R., Bouchenafa O., Ben Maaouia O., Guessasma S. (2019). Mechanosynthesis for kaolinite activation: The impact of the substitution on the mechanical performances of mortar. Powder Technol..

[15] Oleszak D., Kaszuwara W., Wojciechowski S. (1996). Mechanosynthesis of Nd-Fe-B alloys. J. Mater. Sci..

[16] Gaffet E., Le Caër G., Bréchignac C., Houdy P., Lahmani M. (2008). Mechanical Milling. Nanomaterials and Nanochemistry.

[17] Benjamin J.S., Volin T.E. (1974). The mechanism of mechanical alloying. Metall. Trans..

[18] Shoji K., Austin L.G. (1974). A model for Batch Rod Milling. Powder Technol..

[19] El-Eskandarany M.S., Aoki K., Suzuki K. (1990). Rod milling for solid-state formation of Al30Ta70 amorphous alloy powder. J. Less Common Met..

[20] El-Eskandarany M.S. (2015). Mechanical Alloying, Nanotechnology, Materials Science and Powder Metallurgy.

[21] Birringer R. (1989). Nanocrystalline materials. Mater. Sci. Eng. A.

[22] Meyers M.A., Mishra A., Benson D.J. (2006). Mechanical properties of nanocrystalline materials. Prog. Mater. Sci..

[23] Gleiter H. (1989). Nanocrystalline materials. Prog. Mater. Sci..

[24] Suryanarayana C. (2004). Mechanical Alloying and Milling.

[25] Benjamin J.S. (1970). Dispersion strengthened superalloys by mechanical alloying. Metall. Trans..

[26] Hamzaoui R. (2004). Mécanosynthèse et Propriétés Magnétiques D’alliages Fe-Ni.

[27] Malhouroux-Gaffet N., Gaffet E. (1993). Solid state reaction induced by post-milling annealing in the FeSi system. J. Alloys Compd..

[28] Gaffet E., Malhouroux N., Abdellaoui M. (1993). Far from equilibrium phase transition induced by solid-state reaction in the FeSi system. J. Alloys Compd..

[29] Öksüz K.E., Apaydın F., Bozdağ A.E., Çevik M., Özer A. (2015). Phase and Morphological Evaluation of Mechanically Activated Sintered YAG Powders. Procedia Mater. Sci..

[30] Farnè G., Ricciardiello F.G., Podda L.K., Minichelli D. (1999). Innovative milling of ceramic powders: Influence on sintering zirconia alloys. J. Eur. Ceram. Soc..

[31] Zeghmati M., Duverger E., Gaffet E., Tabarrock B., Dost S. (1995). Mechanically Activated Self-Propagating High Temperature Synthesis in the Fe-Al System. Proceedings of the CANCAM, 15th Canadian Congress of Applied Mechanics.

[32] Zhang W., Wang H., Jun H., Yu M., Wang F., Zhou L., Yu G. (2014). Acceleration and mechanistic studies of the mechanochemical dechlorination of HCB with iron powder and quartz sand. Chem. Eng. J..

[33] Andini S., Bolognese A., Formisano D., Manfra M., Montagnaro F., Santoro L. (2012). Mechanochemistry of ibuprofen pharmaceutical. Chemosphere.

[34] Balaz P. (2008). Applied Mechanochemistry. Mechanochem. Nanosci. Miner. Eng..

[35] Grim R.E. (1968). Clay Mineralogy.

[36] Meunier A. (2005). Clays.

[37] Piringer H. (2017). Lime Shaft Kilns. Energy Procedia.

[38] Rodriguez-Navarro C., Ruiz-Agudo E., Luque A., Rodriguez-Navarro A.B., Ortega-Huertas M. (2009). Thermal decomposition of calcite: Mechanisms of formation and textural evolution of CaO nanocrystals. Am. Mineral..

[39] Kumar G.S., Ramakrishnan A., Hung Y.-T. (2007). Lime Calcination. Advanced Physiochemical Treatment Technologies.

[40] Karunadasa K.S.P., Manoratne C.H., Pitawala H.M.T.G.A., Rajapakse R.M.G. (2019). Thermal decomposition of calcium carbonate (calcite polymorph) as examined by in-situ high-temperature X-ray powder diffraction. J. Phys. Chem. Solids.

[41] Karunadasa K.S.P., Manoratne C.H., Pitawala H.M.T.G.A., Rajapakse R.M.G. (2018). The composition, unit cell parameters and microstructure of quartz during phase transformation from α to β as examined by in-situ high-temperature X-ray powder diffraction. J. Phys. Chem. Solids.

[42] Bouchenafa O., Hamzaoui R., Azem L., Bennabi A., Colin J. Manufacturing equivalent Clinker by indirect mechanosynthesis process. Proceedings of the 1st International Conference on Innovation in Low-Carbon Cement and Concrete Technology.

[43] Taylor H.F.W. (1997). Cement Chemistry.

[44] Hughes T.L., Methven C.M., Jones T.G.J., Pelham S.E., Fletcher P., Hall C. (1995). Determining cement composition by Fourier transform infrared spectroscopy. Adv. Cem. Based Mater..

[45] Benosman A.S., Taibi H., Mouli M., Belbachir M. (2004). Valorisation de la spectrométrie infrarouge (FTIR) pour l’analyse qualitative de composes des ciments, argiles, et des mélanges ciment/argile. Communication Science & Technologie.

[46] Sun J., Chen Z. (2018). Influences of limestone powder on the resistance of concretes to the chloride ion penetration and sulfate attack. Powder Technol..

